# The peptidylglycine-α-amidating monooxygenase (*PAM*) gene rs13175330 A>G polymorphism is associated with hypertension in a Korean population

**DOI:** 10.1186/s40246-017-0125-3

**Published:** 2017-11-21

**Authors:** Hye Jin Yoo, Minjoo Kim, Minkyung Kim, Jey Sook Chae, Sang-Hyun Lee, Jong Ho Lee

**Affiliations:** 1 National Leading Research Laboratory of Clinical Nutrigenetics/Nutrigenomics, Department of Food and Nutrition, College of Human Ecology, Yonsei University, 50 Yonsei-ro, Seodaemun-gu, Seoul, 03722 South Korea; 2 Department of Food and Nutrition, Brain Korea 21 PLUS Project, College of Human Ecology, Yonsei University, Seoul, 03722 South Korea; 3 Research Center for Silver Science, Institute of Symbiotic Life-TECH, Yonsei University, Seoul, 03722 South Korea; 4 Department of Family Practice, National Health Insurance Corporation, Ilsan Hospital, Goyang, 10444 South Korea

**Keywords:** Hypertension, Genetic polymorphisms, Genetic association, Peptidylglycine-α-amidating monooxygenase, Atrial natriuretic peptide, LDL atherogenicity

## Abstract

**Background:**

Peptidylglycine-α-amidating monooxygenase (PAM) may play a role in the secretion of atrial natriuretic peptide (ANP), which is a hormone involved in the maintenance of blood pressure (BP). The objective of the present study was to determine whether *PAM* is a novel candidate gene for hypertension (HTN).

**Results:**

A total of 2153 Korean participants with normotension and HTN were included. Genotype data were obtained using the Korean Chip. The rs13175330 polymorphism of the *PAM* gene was selected from the ten single nucleotide polymorphisms (SNPs) most strongly associated with BP. The presence of the G allele of the *PAM* rs13175330 A>G SNP was associated with a higher risk of HTN after adjustments for age, sex, BMI, smoking, and drinking [OR 1.607 (95% CI 1.220–2.116), *p =* 0.001]. The rs13175330 G allele carriers in the HTN group treated without antihypertensive therapy (HTN w/o therapy) had significantly higher systolic and diastolic BP than the AA carriers, whereas the G allele carriers in the HTN group treated with antihypertensive therapy (HTN w/ therapy) showed significantly higher diastolic BP. Furthermore, rs13175330 G allele carriers in the HTN w/o therapy group had significantly increased levels of insulin, insulin resistance, and oxidized low-density lipoprotein (LDL) and significantly decreased LDL-cholesterol levels and LDL particle sizes compared to the AA carriers.

**Conclusion:**

These results suggest that the *PAM* rs13175330 A>G SNP is a novel candidate gene for HTN in the Korean population. Additionally, the *PAM* rs13175330 G allele might be associated with insulin resistance and LDL atherogenicity in patients with HTN.

**Electronic supplementary material:**

The online version of this article (10.1186/s40246-017-0125-3) contains supplementary material, which is available to authorized users.

## Background

Hypertension (HTN) is a significant contributor to the global burden of heart disease, stroke, kidney failure, and premature mortality and disability [1, 2]. HTN is a complex trait that is caused by both genetic and environmental factors [3]. Evidence from family studies indicates that more than 30% of blood pressure (BP) variation can be attributed to genetics [4, 5]. Recently, genome-wide association studies (GWASs) have identified more than 50 single nucleotide polymorphisms (SNPs) associated with an increased risk of HTN [6–8].

The neuroendocrine processing enzyme peptidylglycine-α-amidating monooxygenase (PAM) is highly concentrated in the atrium and may play a role in the secretion of atrial natriuretic peptide (ANP), which is a hormone involved in BP maintenance and fluid homeostasis [9–11]. Indeed, PAM and pro-ANP (the bioactive form of ANP) are the predominant membrane-associated proteins in atrial secretory granules [12]. Because close relationships exist between ANP and BP and between ANP and PAM [9–11], specific *PAM* SNP genotypes in humans may be associated with BP alterations.

Since 2014, the Korean Chip (K-CHIP), which includes 833,535 SNPs and uses an oligomer as a probe, has been developed by the Korea Biobank Array project as a low-cost customized chip that is optimized for genetic studies of diseases and complex traits in Koreans (Additional file 1: Table S1). Since whole-genome sequencing requires very high calculation capacity and cost and commercial chips are designed for Western populations, whose genomic variants differ from Asian populations, the K-CHIP is more suitable for the discovery and identification of Korean population-specific SNPs related to disease occurrence [13, 14]. Although the K-CHIP has only been released recently, several published studies have used the K-CHIP [15, 16]. These studies have gained international recognition; thus, the K-CHIP has been shown to be an appropriate tool for analyzing SNPs associated with diseases in a Korean population.

To the best of our knowledge, this study was the first to investigate HTN-related SNPs using the K-CHIP in a Korean population. Therefore, the objective of the present study was to explore HTN-related SNPs using the K-CHIP, to identify the SNP most strongly associated with BP, and to determine whether *PAM* is a novel candidate gene for HTN among the Korean population.

## Methods

### Study population

A total of 2153 Korean male and female adult participants (male, *n* = 866; female, *n* = 1287; aged 20–86 years; median = 50 years) with nondiabetic normotension (systolic BP < 140 mmHg and diastolic BP < 90 mmHg) and HTN (systolic BP ≥ 140 mmHg or diastolic BP ≥ 90 mmHg) were recruited for this study from the Health Service Center (HSC) during routine checkups at the National Health Insurance Corporation Ilsan Hospital in Goyang, Korea (January 2010–March 2015). Based on the data screened from the HSC, potential subjects with HTN were referred to the Department of Family Medicine or Internal Medicine, where their health and BP were rechecked. Finally, subjects who did not meet the exclusion criteria were all included as study participants. The exclusion criteria were a current diagnosis and/or a history of diabetes, cardiovascular disease, liver disease, renal disease, pancreatitis, cancer, or any life- and health-threatening diseases; pregnancy or lactation; and regular use of any medication except HTN therapy. The aim of the study was carefully explained to all participants, whom provided written informed consent. The Institutional Review Board of Yonsei University and the National Health Insurance Corporation Ilsan Hospital approved the study protocol, which complied with the Declaration of Helsinki.

### Anthropometric measurements

Body weight (UM0703581; Tanita, Tokyo, Japan) and height (GL-150; G-Tech International, Uijeongbu, Korea) were measured in lightly clothed subjects without shoes, and body mass index (BMI) values were calculated (kg/m^2^). Waist circumference was measured directly on the skin at the umbilical level after normal expiration with the subject in an upright standing position. Hip circumference was measured at the protruding part of the hip in standing subjects using a plastic measuring tape with measurements to the nearest 0.1 cm. Waist to hip ratio values were obtained by dividing the waist circumference by the hip circumference. Systolic and diastolic BP were measured using a random-zero sphygmomanometer (HM-1101, Hico Medical Co., Ltd., Chiba, Japan) with appropriately sized cuffs after a rest period of at least 20 min in a seated position. BP was measured three times in both arms. The differences among the three systolic BP measurements were always less than 2 mmHg. Participants were instructed not to smoke or drink alcohol for at least 30 min before each BP measurement.

### Sample collection

Fasting venous blood specimens were collected following an overnight fast of at least 12 h. The samples were collected in EDTA-treated tubes and serum tubes (BD Vacutainer; Becton, Dickinson and Company, Franklin Lakes, NJ, USA). The samples were placed in an ice box that was protected from light within approximately 30 min and then centrifuged (1200 rpm for 20 min at 4 °C) within 3 h to obtain plasma and serum. The plasma and serum aliquots were stored at − 80 °C prior to analysis.

### Serum fasting lipid profiles

The serum fasting triglyceride (TG) and total-cholesterol (TC) levels were measured using enzymatic assays with the TG and CHOL Kits (Roche, Mannheim, Germany), respectively. Serum fasting high-density lipoprotein (HDL)-cholesterol was measured using a selective inhibition method with the HDL-C Plus Kit (Roche, Mannheim, Germany). The resulting color reactions of the assays were monitored using a Hitachi 7600 autoanalyzer (Hitachi, Tokyo, Japan). The Friedewald formula was used to indirectly calculate low-density lipoprotein (LDL)-cholesterol levels as follows: LDL-cholesterol = TC − [HDL-cholesterol + (TG/5)].

### Serum fasting glucose, insulin, and insulin resistance (IR)

The serum fasting glucose level was measured using the hexokinase method with the GLU Kit (Roche, Mannheim, Germany), and the resulting color reaction was monitored with the Hitachi 7600 autoanalyzer (Hitachi, Tokyo, Japan). Serum fasting insulin was measured with an immunoradiometric assay using the Insulin IRMA Kit (DIAsource, Louvain, Belgium), and the resulting color reaction was monitored with an SR-300 system (Stratec, Birkenfeld, Germany). The homeostatic model assessment (HOMA) equation was used to calculate the IR as follows: HOMA-IR = [fasting insulin (μIU/mL) × fasting glucose (mg/dL)]/405.

### Plasma LDL particle size and oxidized (ox)-LDL level

Plasma LDL particles were isolated by sequential flotation ultracentrifugation, and the particle size distribution (1.019–1.063 g/mL) was assessed using a pore-gradient lipoprotein system (CBS Scientific Company, San Diego, CA, USA) on commercially available non-denaturing gels containing a linear 2–16% acrylamide gradient (CBS Scientific Company, San Diego, CA, USA). Latex bead (30 nm)-conjugated thyroglobulin (17 nm), ferritin (12.2 nm), and catalase (10.4 nm) standards were used to measure the relative band migration rates. The gels were scanned using a GS-800 Calibrated Imaging Densitometer (Bio-Rad Laboratories, Hercules, CA, USA). Plasma ox-LDL was estimated using an enzyme immunoassay (Mercodia AB, Uppsala, Sweden), and the resulting color reaction was determined at 450 nm on a Wallac Victor2 multilabel counter (Perkin-Elmer Life Sciences, Boston, MA, USA).

### Affymetrix axiom™ KORV1.0–96 array hybridization and SNP selection

A total of 2167 samples were genotyped according to the manufacturer’s protocol included in the Axiom® 2.0 Reagent Kit (Affymetrix Axiom® 2.0 Assay User Guide; Affymetrix, Santa Clara, CA, USA). Approximately 200 ng of genomic DNA (gDNA) was amplified and randomly fragmented into 25- to 125-base pair (bp) fragments. The initial gDNA amplification was performed in a 40-μL reaction volume containing 20 μL of genomic DNA at a 10 ng/μL concentration and 20 μL of the denaturation master mix. The initial amplification reaction included a 10-min incubation at room temperature; then, the incubated products were amplified with 130 μL of Axiom 2.0 Neutral Soln, 225 μL of Axiom 2.0 Amp Soln, and 5 μL of Axiom 2.0 Amp Enzyme. The amplification reactions were performed for 23 ± 1 h at 37 °C. The amplification products were analyzed in an optimized reaction to amplify fragments between 200 and 1100 bp in length. A fragmentation step reduced the amplified products to segments of approximately 25–50 bp in length, which were end-labeled using biotinylated nucleotides. Following hybridization, the bound target was washed under stringent conditions to remove non-specific background and to minimize the background noise caused by random ligation events. Each polymorphic nucleotide was investigated via a multicolor ligation event conducted on the array surface. After ligation, the arrays were stained and imaged using the GeneTitan MC Instrument (Affymetrix, Santa Clara, CA, USA). The images were analyzed using the Genotyping Console™ Software (Affymetrix, Santa Clara, CA, USA). Genotype data were produced using the K-CHIP, which is available through the K-CHIP consortium. The K-CHIP was designed by the Center for Genome Science at the Korea National Institute of Health (4845–301, 3000–3031).

Samples with the following thresholds were excluded: sex inconsistency, markers with a high missing rate (> 5%), individuals with a high missing rate (> 10%), a minor allele frequency < 0.01, and a significant deviation from the Hardy-Weinberg equilibrium (HWE) (*p* < 0.001). Additionally, SNPs that were in linkage disequilibrium (LD, *r* ^2^ ≥ 0.5) were excluded. The remaining 394,222 SNPs and 2159 samples were included in the subsequent association analysis.

### Statistical analysis

HWE and the associations between SNPs and BP were analyzed with PLINK version 1.07 (http://zzz.bwh.harvard.edu/plink/); the associations were assessed using the linear regression analysis method. Descriptive statistical analyses were conducted using SPSS version 23.0 (IBM, Chicago, IL, USA). For the *PAM* rs13175330 polymorphism, because of the small number of rare allele homozygotes (GG), we pooled heterozygotes (AG) and rare allele homozygotes to increase the statistical power. Logarithmic transformation was used for skewed variables, and data are expressed as the mean ± standard error (SE). A two-tailed *p* value < 0.05 was considered statistically significant. An independent *t* test was performed on continuous variables to compare values between the normotensive group and each hypertensive subgroup and to compare the values between the genotypes within the normotensive group and each hypertensive subgroup. Frequencies were tested with a chi-square test. The association of HTN with a *PAM* rs13175330 genotype was calculated using the odds ratio (OR) [95% confidence intervals (CIs)] of a logistic regression model with adjustments for confounding factors.

## Results

As mentioned above, 394,222 SNPs and 2159 samples were included in the analysis. The ten SNPs that were most strongly associated with BP were selected from the linear regression analysis to assess the association between SNPs and BP. Among them, we identified rs13175330 in the *PAM* gene. The first systolic and diastolic BP-related SNP did not have a reference SNP ID in the SNP database; therefore, we conducted an association analysis using rs13175330, which was the second diastolic BP-related SNP and the seventh systolic BP-related SNP (Additional file 1: Table S2). Among the 2159 genotyped subjects, 6 subjects did not possess a *PAM* variant; thus, only 2153 subjects were included in the final sample.

This was a large study with many samples, and the samples were run on multiple assay plates; thus, we checked inter-and intra-assay coefficient of variability (CV) to reduce multiple assay error although the experiments were not repeated. The mean inter- and intra-assay CV (%) of each variable was as follows (inter-assay CV; intra-assay CV): triglyceride (2.18; 0.95), total-cholesterol (1.19; 1.29), HDL-cholesterol (1.13; 1.16), LDL-cholesterol (0.98; 0.65), glucose (1.01; 0.71), insulin (4.25; 3.55), LDL particle size (2.29; 3.88), and ox-LDL (0.96; 6.78).

### Clinical and biochemical characteristics according to the presence of hypertension

A total of 2153 subjects were divided into a normotensive control group (*n* = 1610) and an HTN group (*n* = 543). The HTN group was stratified according to their antihypertensive therapy [patients with HTN without antihypertensive therapy (HTN w/o therapy), *n* = 377; and patients with HTN with antihypertensive therapy (HTN w/ therapy), *n* = 166]. The clinical and biochemical characteristics of each group are shown in Table 1. Patients in the HTN group and all of the HTN subgroups were significantly older and heavier and had significantly higher systolic and diastolic BP, TG, glucose, insulin, and HOMA-IR indices than the normotensive controls (Table 1). Conversely, the HTN group and all of the HTN subgroups had significantly decreased HDL-cholesterol levels compared with the normotensive controls (Table 1). Ox-LDL was significantly increased in the HTN and HTN w/o therapy groups but not in the HTN w/ therapy group compared to the normotensive controls (Table 1).

**Table 1:** Clinical and biochemical characteristics in the normotensive controls and HTN patient subgroups according to the antihypertensive therapy

	Normotensive controls (*n* = 1610)		HTN group (*n* = 543)					
			Total (*n* = 543)		HTN w/o therapy (*n* = 377)		HTN w/ therapy (*n* = 166)	
Age (years)	48.0	± 0.27	54.4	± 0.50^***^	53.0	± 0.61^***^	57.7	± 0.83^***^
Weight (kg)	63.0	± 0.25	68.1	± 0.51^***^	68.9	± 0.64^***^	66.3	± 0.81^***^
BMI (kg/m^2^)	23.7	± 0.07	25.4	± 0.14^***^	25.4	± 0.17^***^	25.2	± 0.22^***^
Waist (cm)	83.5	± 0.19	87.9	± 0.37^***^	87.9	± 0.46^***^	88.0	± 0.63^***^
Waist hip ratio	0.88	± 0.00	0.90	± 0.00^***^	0.90	± 0.00^***^	0.91	± 0.00^***^
Systolic BP (mmHg)	116.4	± 0.29	138.5	±0.66^***^	145.2	± 0.62^***^	123.2	± 0.79^***^
Diastolic BP (mmHg)	72.7	± 0.22	87.4	± 0.46^***^	91.8	± 0.42^***^	77.4	± 0.66^***^
Triglyceride (mg/dL)^a^	119.6	± 1.84	148.6	± 3.76^***^	151.2	± 4.79^***^	142.8	± 5.72^***^
Total-cholesterol (mg/dL)^a^	198.1	± 0.90	198.3	± 1.54	200.2	± 1.87	193.9	± 2.68
HDL-cholesterol (mg/dL)^a^	53.9	± 0.34	50.4	± 0.55^***^	50.3	± 0.65^***^	50.5	± 1.03^**^
LDL-cholesterol (mg/dL)^a^	121.1	± 0.82	119.1	± 1.42	120.7	± 1.77	115.4	± 2.31
Glucose (mg/dL)^a^	95.6	± 0.51	103.9	± 1.11^***^	103.9	± 1.40^***^	103.9	± 1.73^***^
Insulin (μIU/dL)^a^	9.09	± 0.12	9.84	± 0.25^*^	10.1	± 0.33^*^	9.73	± 0.35^*^
HOMA-IR^a^	2.15	± 0.03	2.55	± 0.09^***^	2.65	± 0.12^***^	2.44	± 0.09^***^
LDL particle size (nm)^a^	23.9	± 0.03	24.0	± 0.05	23.9	± 0.06	24.0	± 0.08
Oxidized LDL (U/L)^a^	46.1	± 0.53	48.3	± 0.97^*^	50.8	± 1.15^***^	43.7	± 1.75

Mean ± SE

*HTN* hypertension, *HTN w/o therapy* HTN group treated without antihypertensive therapy, *HTN w/ therapy* HTN group treated with antihypertensive therapy, *BMI* body mass index, *BP* blood pressure, *HDL* high-density lipoprotein, *LDL* low-density lipoprotein, *HOMA-IR* homeostatic model assessment of insulin resistance

^*^ *p* < 0.05, ^**^ *p* < 0.01, and ^***^ *p* < 0.001 derived from an independent *t* test between the normotensive controls and each HTN subgroup

^a^Tested following logarithmic transformation

### Distribution of the *PAM* rs13175330 A>G polymorphism

The genotype distributions of the *PAM* rs13175330 A>G polymorphism were in HWE in the entire population. Among the 1610 normotensive controls, 1377 subjects (85.5%) had the AA genotype, 228 subjects (14.2%) had the AG genotype, and 5 subjects (0.3%) had the GG genotype. The allele frequency of the G allele was 0.074 in the normotensive controls. Conversely, among the 543 patients with HTN, 434 subjects (79.9%) had the AA genotype, 102 (18.8%) subjects had the AG genotype, and 7 (1.29%) subjects had the GG genotype. The allele frequency of the G allele was 0.107 in the HTN group. The distribution of the *PAM* rs13175330 A>G genotype (*p =* 0.001) and the allele frequencies (*p =* 0.001) in the HTN group differed significantly from the values obtained in the normotensive controls (Additional file 1: Table S3).

### Increased HTN risk associated with the *PAM* rs13175330 A>G polymorphism

Table 2 shows the unadjusted and adjusted odds ratios (OR) for all patients with HTN according to their *PAM* rs13175330 genotype. The presence of the GG genotype of the *PAM* rs13175330 A>G SNP was associated with a higher risk of HTN [OR 4.192 (95% CI 1.325–13.263), *p* = 0.015] (Table 2). The significance of the association remained after adjustments for confounding factors, including age, sex, BMI, smoking, and drinking [OR 7.826 (95% CI 2.228–27.484), *p =* 0.001]. Moreover, the rs13175330 G allele was associated with a higher risk of HTN before [OR 1.484 (95% CI 1.154–1.909), *p* = 0.002] and after adjustments for the confounding factors [OR 1.607 (95% CI 1.220–2.116), *p =* 0.001] (Table 2).

**Table 2:** Unadjusted and adjusted OR for all patients with HTN according to the *PAM* rs13175330 genotypes

*PAM* rs13175330	HTN group (*n* = 543) OR (95% CI)	*p* values
Model 1		
A^*∥*^ compared with G	1.498 (1.186, 1.892)	0.001
AA + AG^*∥*^ compared with GG	4.192 (1.325, 13.263)	0.015
AA^*∥*^ compared with AG + GG	1.484 (1.154, 1.909)	0.002
Model 2		
A^*∥*^ compared with G	1.642 (1.272, 2.121)	< 0.001
AA + AG^*∥*^ compared with GG	7.826 (2.228, 27.484)	0.001
AA^*∥*^ compared with AG + GG	1.607 (1.220, 2.116)	0.001

^*∥*^Reference

*CI* confidence interval, *Model 1* unadjusted, *Model 2* adjusted for age, sex, BMI, smoking, and drinking, *OR* odds ratio, *HTN* hypertension, *PAM* peptidylglycine-α-amidating monooxygenase

### Association between BP and the *PAM* rs13175330 A>G genotype

No significant genotype-related differences were observed among the normotensive controls or HTN subjects treated with/without antihypertensive therapy according to the *PAM* rs13175330 A>G genotype with respect to age, sex, BMI, smoking, and drinking (data not shown). In the normotensive controls, rs13175330 G allele carriers tended to have higher systolic BP than the AA carriers (*p* = 0.056) (Table 3). In the HTN w/o therapy group, rs13175330 G allele carriers had significantly higher systolic BP (*p* = 0.036) and diastolic BP (*p* = 0.048) than the AA carriers. Additionally, the rs13175330 G allele carriers in the HTN w/ therapy group had significantly higher diastolic BP (*p* < 0.001); however, no significant difference in systolic BP between rs13175330 G allele and AA carriers was observed in the HTN w/ therapy group (Table 3).

**Table 3:** Clinical and biochemical characteristics in the normotensive controls and HTN patient subgroups according to the *PAM* rs13175330 genotype

*PAM* rs13175330	Normotensive controls (*n* = 1610)				HTN group (*n* = 543)							
					HTN w/o therapy (*n* = 377)				HTN w/ therapy (*n* = 166)			
	AA (*n* = 1377)		G allele (*n* = 233)		AA (*n* = 305)		G allele (*n* = 72)		AA (*n* = 129)		G allele (*n* = 37)	
Systolic BP (mmHg)	116.2	± 0.31	117.7	± 0.74^*†*^	144.6	± 0.68	147.9	± 1.46^*^	123.2	± 0.90	123.4	± 1.70
Diastolic BP (mmHg)	72.6	± 0.23	73.3	± 0.58	91.4	± 0.45	93.8	± 1.11^*^	75.7	± 0.63	83.3	± 1.67^***^
Triglyceride (mg/dL)^a^	119.4	± 2.02	121.1	± 4.29	148.2	± 5.10	163.9	± 12.7	141.3	± 6.79	148.4	± 9.93
Total-cholesterol (mg/dL)^a^	198.0	± 0.96	198.6	± 2.53	201.7	± 2.06	194.1	± 4.43	193.8	± 3.01	194.5	± 5.97
HDL-cholesterol (mg/dL)^a^	54.0	± 0.37	53.5	± 0.84	50.3	± 0.73	50.4	± 1.45	50.7	± 1.20	49.7	± 1.98
LDL-cholesterol (mg/dL)^a^	121.1	± 0.88	121.1	± 2.20	122.7	± 1.99	112.0	± 3.71^*^	115.5	± 2.66	115.1	± 4.63
Glucose (mg/dL)^a^	95.5	± 0.55	96.5	± 1.47	103.7	± 1.49	104.8	± 3.76	105.7	± 2.08	97.5	± 2.56^*^
Insulin (μIU/dL)^a^	9.07	± 0.13	9.26	± 0.28	9.60	± 0.33	12.1	± 0.99^**^	9.56	± 0.38	10.4	± 0.79
HOMA-IR^a^	2.14	± 0.04	2.21	± 0.08	2.50	± 0.11	3.29	± 0.41^**^	2.45	± 0.11	2.40	± 0.17
LDL particle size (nm)^a^	23.9	± 0.04	24.0	± 0.06	24.0	± 0.07	23.5	± 0.12^**^	24.0	± 0.09	23.9	± 0.17
Oxidized LDL (U/L)^a^	45.9	± 0.56	47.4	± 1.53	49.8	± 1.27	55.0	± 2.70^*^	43.7	± 2.09	43.7	± 2.85

Mean ± SE

*HTN* hypertension, *HTN w/o therapy* HTN group treated without antihypertensive therapy, *HTN w/ therapy* HTN group treated with antihypertensive therapy, *BP* blood pressure, *HDL* high-density lipoprotein, *LDL* low-density lipoprotein, *HOMA-IR* homeostatic model assessment of insulin resistance, *PAM* peptidylglycine-α-amidating monooxygenase

^*†*^ *p* < 0.1, ^*^ *p* < 0.05, ^**^ *p* < 0.01, and ^***^ *p* < 0.001 derived from an independent *t* test within the normotensive controls and each HTN subgroup

^a^Tested following logarithmic transformation

### Lipid profiles, insulin levels, LDL particle sizes, and ox-LDL levels according to the *PAM* rs13175330 A>G genotype

In the HTN w/o therapy group, rs13175330 G allele carriers had significantly higher insulin levels (*p* = 0.001), HOMA-IR indices (*p* = 0.002), and ox-LDL levels (*p* = 0.046) than the AA carriers (Table 3) but significantly lower LDL-cholesterol levels (*p* = 0.039) and smaller LDL particle sizes (*p* = 0.003) than the AA carriers. These genotype effects on the lipid profile, insulin level, LDL particle size, and ox-LDL level were not observed in the normotensive controls or the HTN w/ therapy group (Table 3).

## Discussion

To conduct statistical analysis, we pooled heterozygotes (AG) and rare allele homozygotes (GG) because the number of rare allele homozygotes was too small. Even though there are statistical procedures that can handle unbalanced sample size across compared groups, immoderate unbalanced samples can cause problems [17]. In many cases, a sample size of groups stratified by a genotype is unbalanced due to rare allele frequency. To solve the problem, researchers (1) should anticipate the number of study participants via estimation of genotype frequency according to allele frequency or (2) should balance the number of study participants across groups through a prescreening of genotypes [17]. However, in both cases, if rare allele frequency of a SNP in which researchers are interested is too low, the number of study participants increases too much and the loss of time and cost associated with genotype prescreening will be huge [17]. Therefore, as the other strategy for balancing sample size across the groups, we combined AG and GG genotypes; it is more balanced for statistical purposes [17] rather than analyzing the subjects according to the rs13175330 genotypes (AA vs. AG vs. GG).

The major finding of this study was that the minor G allele frequency of *PAM* rs13175330 A>G was significantly higher in the patients with HTN than in the normotensive controls, suggesting an association between *PAM* rs13175330 A>G and HTN. There are no previous publications regarding a relationship between polymorphisms of *PAM* and HTN development and the effects of PAM dysfunction caused by *PAM* polymorphisms on the risk of HTN; thus, this is the first study to suggest that the *PAM* rs13175330 G allele is related to an increase risk of HTN.

*PAM* rs13175330 A>G is an intronic SNP. Introns have several functions including regulation of alternative splicing and gene expression [18]; via regulating a rate of transcriptional elongation, RNA processing, or RNA turnover, intronic regions can influence RNA levels [19]. Studies support that intronic SNPs do affect RNA splicing [20] and mRNA expression [19, 21]. Finally, Zhou et al. [22] reported that an intronic SNP in *CD44* intron 1 is associated with breast cancer development. Many evidences prove that an intronic SNP can be a novel candidate gene for a certain disease (in case of the present study, HTN) through various mechanisms. Likewise, our data in Tables 2 and 3 support that the *PAM* rs13175330 G allele is associated with a higher risk of HTN development compared with the AA genotype although it is an intronic SNP.

The primary function of the neuroendocrine processing enzyme PAM in the atrium may be to package ANP, a hormone involved in the control of BP and the regulation of sodium and water excretion [10, 11], into atrial secretory granules for storage. Additionally, PAM possibly functions in the presence of activated ANP, which results from the proteolytic processing of pro-ANP [9]. The exact mechanism underlying the association between *PAM* rs13175330 A>G and HTN is unknown; thus, it is difficult to ascertain which function of *PAM* listed in Additional file 1: Table S4 (NCBI gene database; http://www.ncbi.nlm.nih.gov/gene/) is related to BP alteration and HTN development. However, since PAM and pro-ANP are the predominant membrane-associated proteins in atrial secretory granules [12] and PAM plays a role in ANP secretion [9], the *PAM* rs13175330 polymorphism may be involved in the dysregulation of ANP secretion and thus cause HTN. Indeed, the significance of the present observations is underscored by the identification of a human polymorphism in the *PAM* locus that is associated with altered systolic and diastolic BP. The present study showed that normotensive control *PAM* rs13175330 G allele carriers showed a trend toward increased systolic BP, whereas *PAM* rs13175330 G allele carriers in the HTN w/o therapy group had significantly higher systolic and diastolic BP than the AA carriers. Moreover, in the HTN w/ therapy group, rs13175330 G allele carriers also showed significantly increased diastolic BP even though antihypertensive medication significantly lowered systolic and diastolic BP in both AA and G allele carriers compared to those in the HTN w/o therapy group (*p* values of systolic and diastolic BP between AA carriers in the HTN w/o therapy and HTN w/ therapy groups: both *p* < 0.001; *p* values of systolic and diastolic BP between G allele carriers in the HTN w/o therapy and HTN w/ therapy groups: both *p* < 0.001). As shown in Table 2, G allele carriers had a significantly high risk of HTN development; therefore, alteration of BP can be partially explained by PAM dysfunction due to the *PAM* rs13175330 polymorphism.

*PAM* gene polymorphisms affect not only BP but also other HTN-related risk factors. Recently, two missense variants in *PAM* (p.Asp563Gly and p.Ser539Trp) were reported to be associated with a high risk of type 2 diabetes [23]. Additionally, Czyzyk et al. [12] showed that 10-month-old PAM-heterozygous mice had mild but significant glucose intolerance compared to wild-type mice. Although the *PAM* rs13175330 A>G SNP found in this study was different from those SNPs, rs13175330 G allele carriers showed significantly higher insulin and HOMA-IR indices than the AA carriers in the HTN w/o therapy group. Moreover, *PAM* rs13175330 G allele carriers showed significantly smaller LDL particle sizes and higher ox-LDL levels than the AA carriers in the HTN w/o therapy group even though the G allele carriers had significantly lower LDL-cholesterol levels. Thus, the rs13175330 G allele may be associated with worse atherogenicity of LDL-cholesterol. To verify associations between *PAM* genotypes and each variable involved in HTN development (HOMA-IR, LDL particle size, and ox-LDL), we performed a logistic regression analysis (data not shown). The G allele carriers in the HTN group were significantly associated with high HOMA-IR before adjustment for confounding factors including age, sex, BMI, smoking, and drinking [OR 1.110 (95% CI 1.007–1.224), *p* = 0.036]; after adjustment for confounding factors, only tendency was remained [OR 1.094 (95% CI 0.993–1.205), *p* = 0.069]. LDL particle size was significantly small in the G allele carriers before [OR 0.579 (95% CI 0.390–0.861), *p* = 0.007] and after [OR 0.529 (95% CI 0.349–0.800), *p* = 0.003] adjustment. Ox-LDL did not show any association with *PAM* rs13175330 genotypes. In summary, HOMA-IR and LDL particle size are associated with HTN development along with the *PAM* rs13175330 polymorphism. Thus, the increased risk of HTN in the *PAM* rs13175330 G allele carriers can be partially explained by the association between the *PAM* rs13175330 mutation and alteration of glucose tolerance and atherogenicity of LDL-cholesterol.

Taken together, our results indicated a genotype effect from the *PAM* rs13175330 A>G SNP on systolic and diastolic BP, insulin level, the HOMA-IR index, LDL particle size, and ox-LDL level in the HTN w/o therapy group. Since type 2 diabetes [24], decreased LDL particle size [25], and increased ox-LDL level [26] are well-known atherogenic traits related to HTN, these results suggest that the *PAM* gene polymorphism may be involved in HTN development via complex mechanisms, including PAM dysfunction, alterations of glucose tolerance, and atherogenicity of LDL-cholesterol.

Our results share the limitations of cross-sectional observational studies, because we evaluated only associations rather than prospective predictions. Since we verified only the relationship between the *PAM* rs13175330 polymorphism and the risk of HTN, exact mechanisms regarding HTN development by the rs1317530 SNP cannot be fully explained; thus, further studies are needed to demonstrate an association between PAM dysfunction due to the *PAM* rs13175330 polymorphism and HTN. Additionally, we specifically focused on a representative group of Korean subjects in the present study. Therefore, our results cannot be generalized to other ethnic, age, or geographic groups. Moreover, the IR in the HTN group could exaggerate other cardiometabolic syndrome phenotypes and should be considered when interpreting the present findings. Despite these limitations, our results show an interesting association between the *PAM* rs13175330 G allele and an increased risk of HTN.

## Conclusions

Although *PAM* has diverse functions (Additional file 1: Table S4), there are a lack of studies on the association between PAM dysfunction due to *PAM* polymorphisms and diseases. Therefore, the findings in the present study are valuable, as this study reported associations between *PAM* polymorphisms and HTN for the first time. Our study suggests that the *PAM* rs13175330 A>G SNP is a novel candidate gene for HTN among the Korean population. Additionally, the *PAM* rs13175330 G allele may be associated with IR and LDL atherogenicity in patients with HTN. To verify the exact *PAM* rs131753301 polymorphism-related mechanisms underlying HTN development, further studies are required.

## Additional file

Additional file 1: Table S1. Characteristics of the Korean chip. **Table S2.** Top ten SNPs associated with systolic and diastolic BP. **Table S3.** Frequencies of the *PAM* rs13175330 A>G SNP genotypes in the normotensive controls and HTN group based on antihypertensive therapy. **Table S4.** *PAM* gene function. (DOCX 22 kb)

## Abbreviations

ANP: Atrial natriuretic peptide
BMI: Body mass index
bp: Base pair
BP: Blood pressure
CV: Coefficient of variability
gDNA: Genomic DNA
GWAS: Genome-wide association study
HDL: High-density lipoprotein
HOMA: Homeostatic model assessment
HTN: Hypertension
HWE: Hardy-Weinberg equilibrium
IR: Insulin resistance
K-CHIP: Korean Chip
LDL: Low-density lipoprotein
OR: Odds ratio
Ox-: Oxidized
PAM: Peptidylglycine-α-amidating monooxygenase
SNP: Single nucleotide polymorphism
TC: Total cholesterol
TG: Triglyceride

## References

[1] World Health Organization (2013). A global brief on hypertension.

[2] Lim SS, Vos T, Flaxman AD, Danaei G, Shibuya K, Adair-Rohani H (2012). A comparative risk assessment of burden of disease and injury attributable to 67 risk factors and risk factor clusters in 21 regions, 1990-2010: a systematic analysis for the global burden of disease study 2010. Lancet.

[3] Xi B, Cheng H, Shen Y, Zhao X, Hou D, Wang X (2012). Physical activity modifies the associations between genetic variants and hypertension in the Chinese children. Atherosclerosis.

[4] El Shamieh S, Visvikis-Siest S (2012). Genetic biomarkers of hypertension and future challenges integrating epigenomics. Clin Chim Acta.

[5] Van Rijn MJ, Schut AF, Aulchenko YS, Deinum J, Sayed-Tabatabaei FA, Yazdanpanah M (2007). Heritability of blood pressure traits and the genetic contribution to blood pressure variance explained by four blood-pressure-related genes. J Hypertens.

[6] Levy D, Ehret GB, Rice K, Verwoert GC, Launer LJ, Dehghan A (2009). Genome-wide association study of blood pressure and hypertension. Nat Genet.

[7] Padmanabhan S, Melander O, Johnson T, Di Blasio AM, Lee WK, Gentilini D (2010). Genome-wide association study of blood pressure extremes identifies variant near UMOD associated with hypertension. PLoS Genet.

[8] International consortium for blood pressure genome-wide association studies (2011). Genetic variants in novel pathways influence blood pressure and cardiovascular disease risk. Nature.

[9] O'Donnell PJ, Driscoll WJ, Bäck N, Muth E, Mueller GP (2003). Peptidylglycine-alpha-amidating monooxygenase and pro-atrial natriuretic peptide constitute the major membrane-associated proteins of rat atrial secretory granules. J Mol Cell Cardiol.

[10] Thibault G, Amiri F, Garcia R (1999). Regulation of natriuretic peptide secretion by the heart. Annu Rev Physiol.

[11] Sagnella GA (2001). Atrial natriuretic peptide mimetics and vasopeptidase inhibitors. Cardiovasc Res.

[12] Czyzyk TA, Ning Y, Hsu MS, Peng B, Mains RE, Eipper BA (2005). Deletion of peptide amidation enzymatic activity leads to edema and embryonic lethality in the mouse. Dev Biol.

[13] Department of Infectious Disease Control, Korea Centers for Disease Control & Prevention (KCDC). Public health weekly report, PHWR 2015: Vol. 8 No. 29. In: The Korea Biobank Array Project. Korea Centers for Disease Control & Prevention. 2015. http://www.cdc.go.kr/CDC/info/CdcKrInfo0301.jsp?menuIds=HOME001-MNU1154-MNU0005-MNU0037&q_type=&year=2015&cid=64288&pageNum=. Accessed 6 Nov 2017.

[14] Korea Centers for Disease Control & Prevention (KCDC). Korean Chip project. 2017. http://cdc.go.kr/CDC/eng/contents/CdcEngContentView.jsp?cid=74266&menuIds=HOME002-MNU0576-MNU0586. Accessed 6 Nov 2017.

[15] Kim M, Kim M, Yoo HJ, Yun R, Lee SH, Lee JH. Estrogen-related receptor γ gene (*ESRRG*) rs1890552 A>G polymorphism in a Korean population: association with urinary prostaglandin F_2α_ concentration and impaired fasting glucose or newly diagnosed type 2 diabetes. Diabetes Metab. 2017;43:385–8. 10.1016/j.diabet.2016.11.001 28034514

[16] Kim M, Yoo HJ, Kim M, Seo H, Chae JS, Lee SH (2017). Influence of estrogen-related receptor γ (*ESRRG*) rs1890552 A > G polymorphism on changes in fasting glucose and arterial stiffness. Sci Rep.

[17] Roth SM, Roth SM (2007). Genetics primer for exercise science and health. Issues in study design and analysis.

[18] Jo BS, Choi SS (2015). Introns: the functional benefits of introns in genomes. Genomics Inform.

[19] Wang D, Guo Y, Wrighton SA, Cooke GE, Sadee W (2011). Intronic polymorphism in *CYP3A4* affects hepatic expression and response to statin drugs. Pharmacogenomics J.

[20] Wang D, Sadee W (2016). *CYP3A4* intronic SNP rs35599367 (*CYP3A4*22*) alters RNA splicing. Pharmacogenet Genomics.

[21] Xia Z, Yang T, Wang Z, Dong J, Liang C (2014). *GRK5* intronic (CA)_n_ polymorphisms associated with type 2 diabetes in Chinese Hainan Island. PLoS One.

[22] Zhou J, Nagarkatti PS, Zhong Y, Creek K, Zhang J, Nagarkatti M (2010). Unique SNP in *CD44* intron 1 and its role in breast cancer development. Anticancer Res.

[23] Steinthorsdottir V, Thorleifsson G, Sulem P, Helgason H, Grarup N, Sigurdsson A (2014). Identification of low-frequency and rare sequence variants associated with elevated or reduced risk of type 2 diabetes. Nat Genet.

[24] Mehta JL, Rasouli N, Sinha AK, Molavi B (2006). Oxidative stress in diabetes: a mechanistic overview of its effects on atherogenesis and myocardial dysfunction. Int J Biochem Cell Biol.

[25] Maruyama C, Imamura K, Teramoto T (2003). Assessment of LDL particle size by triglyceride/HDL-cholesterol ratio in non-diabetic, healthy subjects without prominent hyperlipidemia. J Atheroscler Thromb.

[26] Witztum JL, Steinberg D (1991). Role of oxidized low density lipoprotein in atherogenesis. J Clin Invest.

