# Supplementary material for: The peptidylglycine-α-amidating monooxygenase (PAM) gene rs13175330 A>G polymorphism is associated with hypertension in a Korean population
Source: Hum Genomics. 2017 Nov 21;11:29. doi: 10.1186/s40246-017-0125-3 (PMC5696732; doi:10.1186/s40246-017-0125-3)
Supplement: Additional file 1: Table S1. — Characteristics of the Korean chip. Table S2. Top ten SNPs associated with systolic and diastolic BP. Table S3. Frequencies of the PAM rs13175330 A>G SNP genotypes in the normotensive controls and HTN group based on antihypertensive therapy. Table S4. PAM gene function. (DOCX 22 kb) [file 40246_2017_125_MOESM1_ESM.docx]

**The peptidylglycine-α-amidating monooxygenase (*PAM*) gene rs13175330 A>G polymorphism is associated with hypertension in a Korean population**

**Additional file**

**Table S1. Characteristics of the Korean chip**

| **Characteristics** | **Description** |
| --- | --- |
| Genomic Coverage | 95% (MAF≥5%)  73% (MAF 1-5%) |
| Number of SNPs | 833,535 |
| SNP Contents | Tagging SNPs*^†^*  Functional SNPs*^‡^* |
| Reproducibility*^§^* | 99.8% |
| Accuracy*^§§^* | 99.7% |

*^†^* Tagging SNPs: Tagging SNPs are based on Asian NGS (next generation sequencing) information (286 Chinese and Japanese information from 1000 genomes project) and Korean population information (over 2,000 individuals).

*^‡^* Functional SNPs: Variants may affect protein’s structural change or protein dysfunction and disease associated variants that already previously known and reported from other research.

*^§^* Reproducibility: Confirming whether acquired SNP information is identical during repeated experiments over 2 times.

*^§§^* Accuracy: Confirming whether acquired SNP information is identical with SNP information that was obtained through other experiments.

**Table S2. Top ten SNPs associated with systolic and diastolic BP.**

| **No.** | **CHR** | **Associated gene** | **SNP** |
| --- | --- | --- | --- |
| **Systolic BP** | | | |
| 1 | 19 | *TPRX1* | - |
| 2 | 3 | *EPHA6* | rs4857055 |
| 3 | 1 | *SRRM1, CLIC4* | rs117559502 |
| 4 | 21 | *LINC00478, C21orf37* | rs116861740 |
| 5 | 12 | *AMN1* | rs142983199 |
| 6 | 18 | *SERPINB7, SERPINB2* | rs62099117 |
| 7 | 5 | ***PAM*** | **rs13175330** |
| 8 | 10 | *ARHGAP22* | rs151307475 |
| 9 | 18 | *LOC100505817, FBXO15* | rs4280345 |
| 10 | 12 | *KRAS, LMNTD1* | rs12828533 |
| **Diastolic BP** | | | |
| 1 | 19 | *TPRX1* | - |
| 2 | 5 | ***PAM*** | **rs13175330** |
| 3 | 1 | *RGS7* | rs1915872 |
| 4 | 22 | *TOP3B, VPREB1* | rs6001482 |
| 5 | 7 | *NOBOX* | rs12539814 |
| 6 | 19 | *SLC5A5* | rs149026664 |
| 7 | 12 | *LINC00936, LINC00615* | rs17192198 |
| 8 | 11 | *NAV2* | rs1559665 |
| 9 | 22 | *PVALB* | rs9607382 |
| 10 | 4 | *LOC101927282, C4orf33* | rs117986095 |

**Table S3. Frequencies of the *PAM* rs13175330 A>G SNP genotypes in the normotensive controls and HTN group based on antihypertensive therapy.**

|  | | ***PAM* rs13175330** | | | | | | | |
| --- | --- | --- | --- | --- | --- | --- | --- | --- | --- |
|  |  | **AA** | | **AG** | | **GG** | | **G allele frequency** | |
|  |  | *n* | % | *n* | % | *n* | % | *n* | % |
| **Normotensive controls (*n*=1,610)** | | 1,377 | 85.5 | 228 | 14.2 | 5 | 0.3 | 238 | 7.4 |
|  | Total (*n*=543) | 434 | 79.9 | 102 | 18.8 | 7 | 1.3 | 116 | 10.7 |
| **HTN group (*n*=543)** | HTN w/o therapy (*n*=377) | 305 | 80.9 | 68 | 18.0 | 4 | 1.1 | 76 | 10.1 |
|  | HTN w/ therapy (*n*=166) | 129 | 77.7 | 34 | 20.5 | 3 | 1.8 | 40 | 12.0 |
|  | Total | 0.001 | | | | | | 0.001 | |
| ***p*-values compared with controls** | HTN w/o therapy | 0.022 | | | | | | 0.014 | |
|  | HTN w/ therapy | 0.002 | | | | | | 0.003 | |

A chi-squared test was used to calculate the *p*-values.

**Table S4. *PAM* gene function.**

| **Function of *PAM*** |
| --- |
| L-ascorbic acid binding |
| Calcium ion binding |
| Copper ion binding |
| Peptidylamidoglycolate lyase activity |
| Peptidylglycine monooxygenase activity |
| Protein binding |
| Protein kinase binding |
| Zinc ion binding |

NCBI gene database (http://www.ncbi.nlm.nih.gov/gene/)
